# U-Shaped Association between Maternal Hemoglobin and Low Birth Weight in Rural Bangladesh

**DOI:** 10.4269/ajtmh.21-0268

**Published:** 2021-11-29

**Authors:** Rebecca M. Carpenter, Sk Masum Billah, Genevieve R. Lyons, Md Shahjahan Siraj, Qazi S. Rahman, Vanessa Thorsten, Elizabeth M. McClure, Rashidul Haque, William A. Petri

**Affiliations:** ^1^ Division of Infectious Diseases and International Health, University of Virginia School of Medicine, Charlottesville, Virginia;; ^2^ The International Center for Diarrhoeal Disease and Research, Dhaka, Bangladesh;; ^3^ The University of Sydney School of Public Health, Sydney, Australia;; ^4^ Department of Public Health Sciences, University of Virginia, Charlottesville, Virginia;; ^5^ RTI International, Research Triangle Park, North Carolina

## Abstract

Low birth weight (LBW) is associated with a higher risk of neonatal mortality and the development of adult-onset chronic disease. Understanding the ongoing contribution of maternal hemoglobin (Hgb) levels to the incidence of LBW in South Asia is crucial to achieve the World Health Assembly global nutrition target of a 30% reduction in LBW by 2025. We enrolled pregnant women from the rural Tangail District of Bangladesh in a Maternal Newborn Health Registry established under The Global Network for Women’s and Children’s Health Research. We measured the Hgb of pregnant women at enrollment and birth weights of all infants born after 20 weeks gestation. Using logistic regression to adjust for multiple potential confounders, we estimated the association between maternal Hgb and the risk of LBW. We obtained Hgb measurements and birth weights from 1,665 mother–child dyads between July 2019 and April 2020. Using trimester-specific cutoffs for anemia, 48.3% of the women were anemic and the mean (±SD) Hgb level was 10.6 (±1.24) g/dL. We identified a U-shaped relationship where the highest risk of LBW was seen at very low (< 7.0 g/dL, OR = 2.00, 95% CI = 0.43–7.01, *P* = 0.31) and high (> 13.0 g/dL, OR = 2.17, 95% CI = 1.01–4.38, *P* = 0.036) Hgb levels. The mechanisms underlying this U-shaped association may include decreased plasma expansion during pregnancy and/or iron dysregulation resulting in placental disease. Further research is needed to explain the observed U-shaped relationship, to guide iron supplementation in pregnancy and to minimize the risk of LBW outcomes.

## INTRODUCTION

Low birth weight (LBW) continues to be a pressing global health concern as approximately 20 million babies are born each year with a weight of < 2,500 grams.^1^ This threshold for LBW was established by epidemiologic studies that demonstrated that babies with a birth weight of less than 2,500 grams were 20 times more likely to die in infancy.^2^^,^^3^ LBW is associated with 80% of all neonatal deaths and has been linked to the development of stunting and adult-onset-noncommunicable disease through malnutrition and fetal programming in utero.^1^^,^^4^^,^^5^

In 2012, the WHO set global nutrition targets under the Millennium Development Goals (MDGs) that include a 30% reduction in LBW by the year 2025.^1^^,^^6^ These goals have brought attention to South Asia as about half of all LBW infants are born in India and Bangladesh. National LBW surveys conducted in Bangladesh in 2003–2004 and again in 2015 document significant progress in reducing the incidence of LBW in the region from 36% in 2003–2004 to 22.6% in 2015.^3^ This progress has been attributed to improvement in socioeconomic conditions and widespread implementation of routine iron-folate supplementation.^3^ Despite this progress, further work is needed to reduce LBW in South Asia to a rate comparable with the 6% of all births seen in many developed nations.^3^^,^^6^

Adequate iron stores are required during pregnancy for expansion of the maternal red cell mass that supports the growing placenta and fetus. Inadequate iron stores at the start of pregnancy place women at risk for the development of iron-deficiency anemia.^7^^,^^8^ For this reason, universal preventative iron supplementation is routinely included in prenatal care.^9^^,^^10^ This practice is supported by a positive correlation between iron supplementation and birth weight.^11^^,^^12^ Anemia, however, which is often used as a proxy for iron deficiency, is inconsistently associated with LBW.^13^ The national LBW survey in Bangladesh in 2015 found no association between hemoglobin (Hgb) and birth weight whereas a large systematic review and meta-analysis conducted in 2016 found the anemia-attributable proportion of LBW in low-income countries was 25%.^3^^,^^14^^,^^15^ Some studies have found an association between only severe anemia (< 7.0 g/dL) and LBW, whereas others document an increased risk of LBW at any Hgb level less than 11.0 g/dL, particularly when combined with a low maternal body mass index (BMI).^16^^,^^17^

Here, we report findings on the association of maternal Hgb with risk of LBW from an ongoing Maternal and Newborn Health Registry. It is important to further clarify the relationship between anemia and LBW in Bangladesh to bolster progress toward meeting the global nutrition target of a 30% reduction in LBW by the year 2025.

## MATERIALS AND METHODS

### Data sources and sampling procedure.

**Figure 1.**
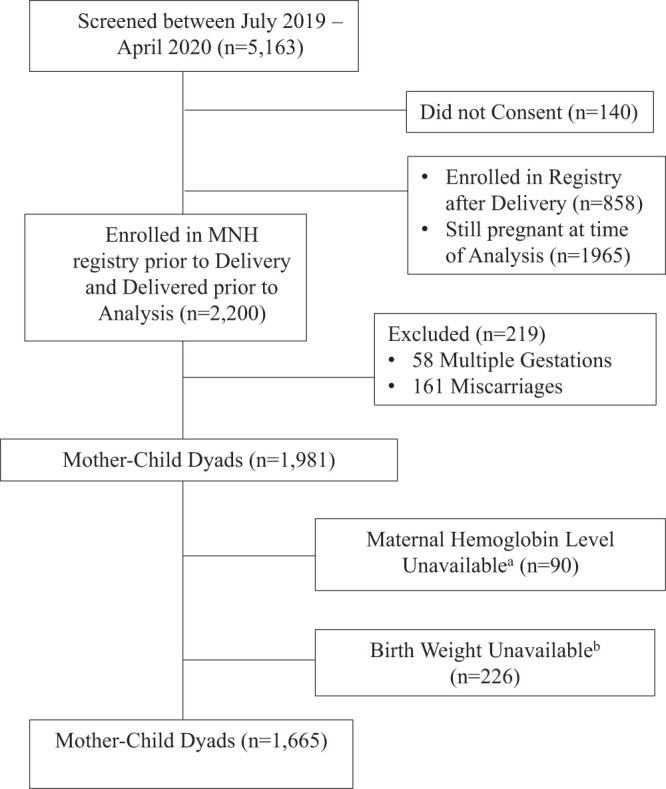
Diagram of maternal newborn health registry enrollment in Bangladesh, 2019–2020. ^a^Of the eligible population for this analysis, 90 women refused to allow a hemoglobin level; ^b^Accurate birth weight information was not recorded for 226 neonates (107 of these had birth weight estimates recorded, 63 had birth weights taken after 14 days of life, and 56 neonates had no birth weight recorded).

This was a population-based study that is part of the National Institute of Child Health Global Network Maternal Newborn Health Registry (MNHR) (ClinicalTrials.gov Identifier: NCT01073475.) MNHR is a multisite, prospective, ongoing, and active surveillance system that is tracking pregnancies and births in defined geographic communities (clusters), each with approximately 300–400 deliveries per year. The Bangladesh site is located in the Tangail District of Bangladesh and is composed of 12 study clusters each with 17,500–19,500 people. Through a bimonthly house-to-house surveillance, pregnant women were identified and 99.7% of all eligible pregnant women were enrolled upon providing consent to participate. Mother–child dyads were followed up at birth (within 72 hours) and at 42 days postpartum for collection of maternal and newborn outcomes. Details of the registry are described elsewhere.^18^^,^^19^ Pregnant women who were screened between July 2019 and April 2020 were included in this analysis as presented in Figure 1.

### Outcome variable.

LBW, the main outcome variable, was dichotomized as yes = 1 (baby born with birth weight < 2,500 g) or no = 0 (otherwise). Birth weights were obtained in the field by trained personnel using an AWS H-110 (American Weigh Scales, Inc., Cumming, GA) digital hanging scale, calibrated daily. A large number of newborns in this region of Bangladesh are born at home or in small healthcare facilities, which presents a major challenge to collecting accurate birth weight information.^20^ In this analysis, 226 newborns were excluded because of no reliable birth weight (Figure 1). Of the analyzed birth weights, over 50% were taken on the day of birth, 88% within the first 3 days of life, and 100% before 2 weeks postdelivery. Birth weights taken on day of life 1–13 were adjusted according to expected neonatal weight loss to reduce the risk of overreporting LBW because of the delay in birth weight measurements.^21^

### Explanatory variables.

Blood Hgb, the main explanatory variable, was collected in the field by trained personnel using a point of care hemoglobinometer (HemoCue 301, HemoCue AB, Ängelholm Sweden). The HemoCue 301 analyzer has shown moderate agreement of Hgb level estimation when compared with the gold standard hematology autoanalyzer.^22^ Of the 1,981 eligible mother–child dyads, 90 were excluded because the mother refused a Hgb measurement (Figure 1). Measurements were used as both a continuous and discrete variable categorized into severe anemia, moderate anemia, mild anemia, normal Hgb, and high Hgb. Anemia was defined according to the WHO and the U.S. CDC using trimester-specific cutoffs, which control for normal plasma expansion during pregnancy.^23^ These cutoffs use Hgb < 11.0 g/dL in the first and third trimester and Hgb < 10.5 g/dL in the second trimester as the threshold for anemia.^23^ Trimester of pregnancy at the time of Hgb measurement was determined using the last menstrual period (LMP), which is the most accurate method of determination given low early ultrasound coverage in the rural setting.

The sociodemographic and obstetric characteristics were considered as explanatory variables of occurrence and nonoccurrence of LBW in newborns. The selection process to identify these explanatory variables drew on a range of studies carried out to assess the magnitude of LBW and to identify its determinants.^1^^,^^2^^,^^20^^,^^46^ Clinically significant cut points were used to create categorical variables for maternal age, BMI, and parity. Locally weighted scatterplot smoothing (LOESS), a nonparametric method for fitting a smooth curve to data points, was used to determine appropriate cut points to convert inter-delivery interval to a categorical variable. A composite score using principal component analyses of household assets was used to determine socioeconomic status. A complete list of explanatory variables is presented in Table 1. Given the number of missing prenatal Hgb and birth weight measurements in this dataset, we explored the possibility of systematic differences in explanatory variables between the group analyzed (*N* = 1,665) and the group with missing key variables (*N* = 316). All variables found to have an association with the LBW outcome of interest on univariate analysis were included in this exploration and presented in Table 2.

**Table 1:** Sociodemographic and obstetric characteristics of women–child dyads

Variable	Characteristics	Frequency (%) (*N* = 1665)
Maternal age (years)*	Less than 16	78 (4.7)
	16–35	1,522 (91.4)
	Greater than 35	60 (3.6)
Maternal education	No formal schooling	100 (6.0)
	Primary	566 (34.0)
	Secondary	895 (53.8)
	University+	104 (6.2)
Maternal body mass index (kg/m^2^)*	Underweight	125 (7.5)
	Normal	968 (58.1)
	Overweight	571 (34.3)
Socioeconomic status	Low	149 (8.9)
	Medium	1,301 (78.1)
	High	215 (12.9)
Type of household fuel	Dung/Wood/Charcoal/Straw/Shrubs/Grass	1,476 (88.6)
	LPG/Electricity	189 (11.4)
Antenatal visits	≥ 4 visits	189 (11.4)
	< 4 visits	1,476 (88.6)
Evidence of hypertensive disease*	Yes	81 (4.9)
	No	1,580 (94.9)
Parity	Nulliparous	648 (38.9)
	One–Two	908 (54.5)
	Greater than two	109 (6.5)
Inter-delivery interval	< 40 months	228 (13.7)
	40–70 months	287 (17.2)
	> 70 months	469 (28.2)
	Nulliparous	681 (40.9)
Iron Supplementation*†	Yes	1,371 (82.3)
	No	293 (17.6)
Delivery mode	Vaginal delivery	747 (44.9)
	C-section	918 (55.1)
Season of delivery	Summer (March–October)	556 (33.4)
	Winter (November–February)	1,109 (66.6)
Sex of the baby	Female	821 (49.3)
	Male	844 (50.7)
Trimester of hemoglobin measurement‡	First trimester	195 (11.7)
	Second trimester	749 (45.0)
	Third trimester	721 (43.3)

*Less than 1% of data missing.

†Iron supplementation reported at any time during pregnancy for any duration, note that iron supplementation in this population is common but rarely achieves an adequate dose of 180 tablets during pregnancy.^45^

‡Trimester of hemoglobin measurement determined using last menstrual period (LMP), which is the most accurate method of gestational age dating in this population where ultrasound is not readily available.

**Table 2:** Analysis of systematic differences between included and excluded women–infant dyads

Variable	Characteristics	Excluded (*N* = 316)	Included (*N* = 1665)	*P* value
Maternal age (years)*	Less than 16	5 (1.6)	78 (4.7)	0.0292
	16–35	292 (92.4)	1,522 (91.4)	
	Greater than 35	15 (4.7)	60 (3.6)	
Maternal education	No formal schooling	33 (10.4)	100 (6.0)	0.00654
	Primary	120 (38.0)	566 (34.0)	
	Secondary	147 (46.5)	895 (53.8)	
	University+	16 (5.1)	104 (6.2)	
Maternal body mass index (kg/m^2^)*	Underweight	29 (9.2)	968 (58.1)	0.115
	Normal	193 (61.1)	125 (7.5)	
	Overweight	89 (28.2)	571 (34.3)	
Socioeconomic status	Low	40 (12.7)	149 (8.9)	0.0516
	Medium	244 (77.2)	1,301 (78.1)	
	High	31 (9.8)	215 (12.9)	
Type of household fuel	Dung/Wood/Charcoal/Straw/Shrubs/Grass	285 (90.2)	1,476 (88.6)	0.483
	LPG/Electricity	31 (9.8)	189 (11.4)	
Inter-delivery interval	< 40 months	44 (13.9)	228 (13.7)	0.723
	40–70 months	54 (17.1)	287 (17.2)	
	> 70 months	98 (31.0)	469 (28.2)	
	Nulliparous	120 (38.0)	681 (40.9)	
Delivery mode*	Vaginal delivery	239 (75.6)	747 (44.9)	< 0.0001
	C-section	75 (23.7)	918 (55.1)	
Sex of the baby*	Female	156 (49.4)	821 (49.3)	0.751
	Male	153 (48.4)	844 (50.7)	

* Less than 1% of data missing.

### Statistical analysis.

Data were entered and calculated fields were determined at the study site before secure transmission to the central data center (RTI International) where further data quality checks were performed before statistical analyses. Maternal characteristics were examined using descriptive statistics. Crude associations between maternal characteristics and LBW were assessed using χ^2^, Fisher’s exact tests, and *t*-tests as appropriate. In bivariate analysis, we used χ^2^ and Fisher’s exact for categorical variables to examine whether the outcome was associated with each of the explanatory variables. Independent variables identified in univariate models to be associated with LBW (*P* < 0.1) were included in a multivariable binomial logistic regression. A *P* value of < 0.05 was considered significant when interpreting associations identified by the multivariate analysis. Finally, we used a LOESS to visualize the relationship between maternal Hgb concentration and probability of LBW.

All statistical analyses were conducted in R (version 4.0.3, https://cran.r-project.org/). The MNHR study was reviewed and approved by the ICDDR,B’s (International Centre for Diarrhoeal Disease Research, Bangladesh) Research Review and Ethical Review Committees and the Institutional Review Boards of the corresponding U.S. partners (University of Virginia and RTI International). All women provided informed consent before participation.

## RESULTS

Between July 2019 and April 2020, 5,163 women–child dyads were screened for eligibility. Of those screened, 140 did not provide consent to participate in the MNHR. At the time of the analysis, 1,965 women had not yet delivered and 161 suffered miscarriages. The 58 women with multiple gestations were excluded. Of those remaining, 1,665 mother–child dyads were ultimately included in this study as they had both a Hgb measurement taken during pregnancy and a birth weight measured within the first 2 weeks of life (Figure 1). Over half of these women were enrolled in the MNHR within the first or second trimester (≤ 27 weeks gestation) of pregnancy.

The mean maternal Hgb level in this cohort was 10.6 g/dL with a standard deviation of 1.24 g/dL. The prevalence of anemia was 48.3% and 46% of all anemia cases fell into the category of mild anemia. Only 0.7% of these women exhibited a severe anemia (< 7.0 g/dL). Maternal sociodemographic and obstetric characteristics are displayed in Table 1.

**Figure 2.**
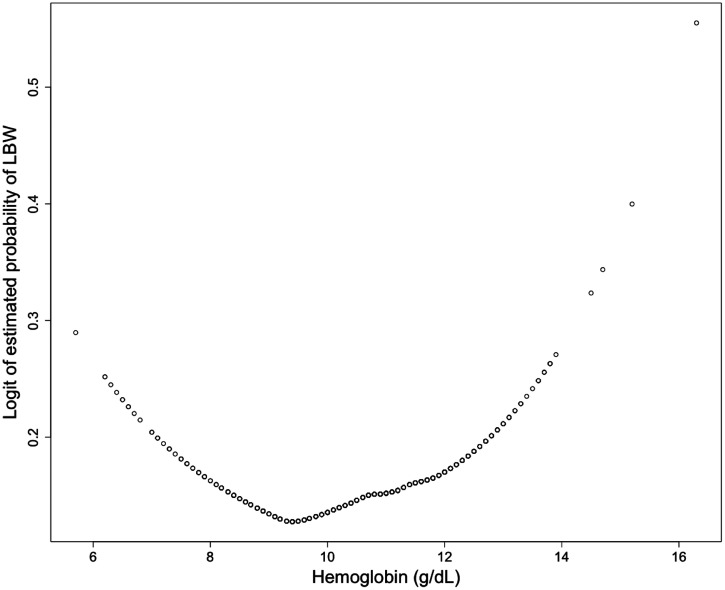
U-shaped association of hemoglobin with low birth weight (LBW) outcome. Note: Locally weighted scatterplot smoothing (LOESS) plot of LBW outcome depicting the logit of the estimated probabilities of LBW at given hemoglobin levels, binomial variables are plotted at 0 for normal birth weight (NBW) and 1 for LBW. The current figure is truncated for better viewing of the LOESS curve.

Infant birth weight outcomes by maternal Hgb category are displayed in Table 3. The prevalence of LBW was 15.3%. We noted that a high maternal Hgb (> 13 g/dL) was associated with a 2-fold higher risk of LBW when compared with a normal Hgb (OR = 2.17, 95% CI = 1.01–4.38, *P* = 0.0362). A LOESS plot of Hgb versus LBW revealed a U-shaped relationship with the lowest risk of LBW associated with Hgb concentrations between 9.0 and 11.0 g/dL (Figure 2). Although no statistically significant association was found between severe anemia (Hgb < 7.0) and risk of LBW, this finding is limited by the small sample size of only 11 severely anemic women (OR = 2.00, 95% CI = 0.43–7.01, *P* = 0.311).

**Table 3:** Low birth weight risk factors

				Univariate model	Multivariate model		
Variable	Characteristics	LBW*	NBW*	*P* value†	AOR‡	(95% CI)‡	*P* value‡
Hemoglobin (g/dL)	Severe anemia	3 (1.2)	8 (0.6)	0.0942	2.73	0.57–10.20	0.159
	Moderate anemia	59 (23.1)	364 (25.8)		0.96	0.63–1.45	0.833
	Mild anemia	52 (20.4)	318 (22.6)		Ref	Ref	Ref
	Normal hemoglobin	130 (51.0)	693 (49.1)		1.11	0.78–1.60	0.573
	High Hemoglobin	11 (4.3)	27 (1.9)		2.28	1.01–4.86	0.0385*
Sex of the baby	Male	106 (41.6)	738 (52.3)	0.00195	Ref	Ref	Ref
	Female	149 (58.4)	672 (47.7)		1.49	1.13–1.97	0.00525*
Mode of delivery	Vaginal Delivery	138 (54.1)	609 (43.2)	0.00158	Ref	Ref	Ref
	Cesarean Delivery	117 (45.9)	801 (56.8)		0.74	0.56–0.99	0.0420*
Season of delivery	Summer (Mar.–Oct.)	76 (29.8)	480 (34.0)	0.212	–	–	–
	Winter (Nov.–Feb.)	179 (70.2)	930 (66.0)		–	–	–
Iron supplementation	Yes	207 (81.2)	1,164 (82.6)	0.642	–	–	–
	No	48 (18.8)	245 (17.4)		–	–	–
Maternal body mass index (kg/m^2^)	Underweight (< 18.5)	27 (10.6)	98 (7.0)	0.000604	1.29	0.79–2.04	0.289
	Normal (18.5–24.9)	165 (64.7)	803 (57.0)		Ref	Ref	Ref
	Overweight (≥ 25)	62 (24.3)	509 (36.1)		0.67	0.48–0.93	0.0170*
Maternal age	< 16	22 (8.6)	56 (4.0)	0.00915	1.91	1.08–3.28	0.0219*
	16–35	224 (87.8)	1,298 (92.1)		Ref	Ref	Ref
	≥ 35	8 (3.1)	52 (3.7)		0.71	0.30–1.50	0.407
Maternal education	No Formal Schooling	25 (9.8)	75 (5.3)	0.0261	Ref	Ref	Ref
	Primary	87 (34.1)	479 (34.0)		0.53	0.32–0.91	0.00182*
	Secondary	132 (51.8)	763 (54.1)		0.51	0.30–0.88	0.0125*
	University+	11 (4.3)	93 (6.6)		0.47	0.20–1.07	0.0794
Socioeconomic status	Low	24 (9.4)	125 (8.9)	0.0534	Ref	Ref	Ref
	Medium	210 (82.4)	1,091 (77.4)		1.14	0.71–1.89	0.595
	High	21 (8.2)	194 (13.8)		0.83	0.42–1.63	0.592
Household fuel	LPG/Electricity	16 (6.3)	173 (12.3)	0.00502	Ref	Ref	Ref
	Dung/Wood/Charcoal/ Straw/Shrubs/Grass	239 (93.7)	1,237 (87.7)		1.72	1.00–3.14	0.0620
Evidence of hypertensive disease	No	239 (93.7)	1,341 (95.1)	0.268	–	–	–
	Yes	16 (6.3)	65 (4.6)		–	–	–
Inter-delivery interval	< 40 months	24 (9.4)	204 (14.5)	0.0926	Ref	Ref	Ref
	40–70 months	39 (15.3)	248 (17.6)		1.37	0.79–2.42	0.262
	> 70 months	77 (30.2)	392 (27.8)		1.63	1.00–2.74	0.0570
	Nulliparous	115 (45.1)	566 (40.1)		1.60	1.00–2.67	0.0583
Number of antenatal visits	≥ 4 visits	31 (12.2)	158 (11.2)	0.739	–	–	–
	< 4 visits	224 (87.8)	1,252 (88.8)		–	–	–
Parity	Nulliparous	109 (42.7)	539 (38.2)	0.397	–	–	–
	One–Two	130 (51.0)	778 (55.2)		–	–	–
	Greater than Two	16 (6.3)	93 (6.6)		–	–	–

* Low birth weight (LBW), normal birth weight (NBW).

† Significant *P* values represent a crude association between explanatory variable and birth weight outcomes based on χ^2^ and Fisher exact tests for each variable separately.

‡ Independent variables identified in univariate models to be associated with LBW (using cutoff of < 0.1) were included in a multivariable logistic regression model with a binomial distribution assumption and log-link accounting for potential confounders. Adjusted odds ratio (AOR), 95% CI, and *P* values reported here.

In bivariate analysis, maternal BMI, age, education, socioeconomic status, sex of the infant, mode of delivery, inter-delivery interval, and type of household fuel were also associated with LBW status (Table 3). Recognizing that significant confounding may exist with these covariates, we used multivariable modeling to control for the impact of these variables (Table 3). Covariates associated with the risk of LBW in this multivariate model include sex of the infant, mode of delivery, maternal BMI, age, and education. Using the bottom category on the U-shaped Hgb curve, mild anemia (Hgb 10.0–11.0 g/dL) as the reference, the risk of LBW was associated with a high Hgb of > 13.0 g/dL (AOR = 2.28, 95% CI = 1.01–4.86, *P* = 0.0385). Although the sample size was too small to reach statistical significance, a Hgb level < 7.0 g/dL was associated with a 2.7-fold risk of LBW (AOR = 2.73, 95% CI = 0.57–10.20, *P* = 0.159). Moderate anemia was not significantly associated with LBW outcomes.

## DISCUSSION

The results from this study suggest that high maternal Hgb concentration (> 13 g/dL) is associated with a 2-fold increase in risk of LBW. On the other end of the spectrum, severe anemia (Hgb < 7 g/dL) may be associated with a similar risk of LBW but the relationship in this cohort was not significant likely due to small numbers (*N* = 11) in this category. The prevalence of maternal anemia in Bangladesh is higher in this cohort than the global average previously described by the WHO, 48.3% versus 42% globally.^24^ Despite this high rate of maternal anemia, the majority of anemic women (mild-moderate anemia) face no increased risk of LBW outcomes in this cohort of 1,665 mother–child dyads.

The literature regarding the impact of maternal anemia on LBW outcomes has not achieved a clear consensus. Early studies found a direct correlation between low Hgb levels and LBW babies.^9^^,^^14^^,^^15^^,^^23^^,^^25^ Other studies have found no association between mild-moderate anemia and LBW.^15^^,^^26^ For example, a large study of over 70,000 mother–child dyads in India found an association between LBW and maternal anemia at all levels.^16^ Another cohort study in India and Pakistan found an association between only severe maternal anemia and LBW.^17^ The most recent national LBW survey in Bangladesh found no association at all between maternal anemia and LBW outcomes.^3^ Other studies have noted an association between LBW and maternal anemia detected during the first or second trimester of pregnancy but not during the third trimester.^27^^,^^28^ These disparities in study findings likely have to do with the multidimensional causation of maternal anemia where not all types of anemia contribute to LBW in the exact same manner.^24^

The most common cause of anemia in pregnant women is iron-deficiency anemia and it is often assumed that over 50% of all cases of maternal anemia in high-prevalence regions like Bangladesh can be attributed to iron deficiency alone.^29^ Iron deficiency has been independently associated with LBW outcomes because the mother’s body does not have sufficient iron stores to adequately expand the red blood cell mass and support the growth of the placenta and developing fetus.^7^^,^^9^^,^^30^ This is especially true for iron deficiency before conception and early in the first trimester.^11^^,^^14^^,^^27^^,^^28^ Routine iron supplementation and nutrition education has been shown to effectively reduce the incidence of LBW in iron-deficient populations.^12^^,^^31^ However, the impact of anemia on LBW may not be as robust in places where the prevalence of iron deficiency is not common as has been suggested in Bangladesh.^24^^,^^32^ The high rate of mild to moderate anemia in this cohort could be secondary to a condition such as thalassemia rather than iron deficiency. Of note, thalassemia-related anemia may not have as large an impact on LBW rates as iron-deficiency anemia.^30^^,^^33^

An important finding of this study is the documentation of a U-shaped relationship between maternal Hgb levels and LBW infant outcomes. This relationship has only recently been described in low- and middle-income countries where many variables impact infant birth weight.^34^^–^^38^ Most recently, a study involving 130,888 pregnant women across Pakistan and India found that high and low maternal Hgb concentrations are related to both LBW and neonatal mortality.^34^

High maternal Hgb levels have been associated with preeclampsia, preterm delivery, and fetal growth restriction, but it is still uncertain if these outcomes are caused by high maternal iron levels or by failure of the plasma volume to expand appropriately.^39^^–^^41^ During pregnancy, a woman’s blood plasma will increase by up to 50%, whereas the red blood cell mass only increases by about 25%.^42^ This hemodilution leads to a relative anemia in most pregnancies. High Hgb levels can be a sign that the plasma volume has not expanded appropriately in women with pregnancy-induced hypertension, preeclampsia, and fetal growth restriction.^36^^,^^41^ However, a study of more than 57,000 pregnancies in Norway found that increasing Hgb levels were associated with lower placental weight and impaired fetal growth in women with and without preeclampsia suggesting another mechanism may be involved apart from failed plasma expansion.^43^ There is also evidence that a primary elevation in iron levels could cause adverse outcomes in pregnant women via damage to the placenta. High Hgb levels have been associated with placental infarction, which could be caused by an increase in oxidative stress.^44^ Elevated iron levels also have been shown to inhibit the uptake of trace elements such as Zn, which are necessary for a healthy pregnancy and fetal growth.^44^

Further research is needed to understand the causal link between high Hgb levels and adverse pregnancy outcomes, including LBW. Although many women did not routinely take their iron supplements, 34 out of 38 of the women with a Hgb > 13.0 g/dL in this cohort were receiving iron supplementation. It is therefore important to delineate the role, if any, of maternal iron levels in the pathophysiology of hemoconcentration-associated adverse birth outcomes to ensure that iron supplementation does not inadvertently cause harm. Iron supplementation is routinely recommended for pregnant women with normal Hgb levels as it is possible to be pre-anemic and iron deficient. However, it is also possible to be anemic and iron sufficient, as has been documented in Bangladesh.^24^^,^^32^ It is imperative to understand the impact of these routine iron supplementation programs so as to not cause any harm with these interventions.

## STRENGTHS AND LIMITATIONS

The bimonthly house-to-house surveillance, and enrollment of eligible pregnant women into the MNHR highlights both a strength and a limitation of this study. Although the registry achieves an almost 100% catchment and enrollment of pregnant women in this rural area, a considerable number (*N* = 316) of eligible maternal–child dyads were excluded from this analysis due to missing key variables. Given the major challenges to obtaining these variables in a rural setting with limited antenatal care and a large number of home deliveries, the current study performed well when contrasted to comparable studies where as many as 71% of newborns show no birth information.^20^ However, as this limitation could not be avoided, we have explored the possibility of systemic differences between the included and excluded groups (Table 2). Compared with those who were excluded, the included group had a slightly higher percentage of educated women and a slightly larger percentage of women in the less than 16 years of age category. These two variables might be expected to have an opposite impact on LBW outcomes and would not be expected to impact the U-shaped relationship of maternal Hgb to the risk of LBW. Other limitations of the study include that Hgb measurements were taken at different times during pregnancy and that some of the Hgb categories had a very small number of subjects.

## CONCLUSION

This study demonstrated an overall low rate of severe anemia in this cohort and revealed no significant increase in the risk of LBW in pregnant women with mild-moderate anemia. In contrast, high maternal Hgb values (> 13 g/dL) were associated with an increased risk of neonatal LBW. These findings suggest that iron-deficiency anemia may not be a significant contributor to the high rate of LBW seen in this population. Further research is needed to understand the U-shaped association of maternal Hgb with LBW outcomes, particularly the biological mechanism underlying the association between adverse outcomes and high maternal Hgb values. Understanding this association will give further guidance to the benefits and possible dangers of routine universal iron supplementation during pregnancy.
